# Electrical Potential of Acupuncture Points: Use of a Noncontact Scanning Kelvin Probe

**DOI:** 10.1155/2012/632838

**Published:** 2012-12-20

**Authors:** Brian J. Gow, Justine L. Cheng, Iain D. Baikie, Ørjan G. Martinsen, Min Zhao, Stephanie Smith, Andrew C. Ahn

**Affiliations:** ^1^Osher Center for Integrative Medicine, Brigham and Women's Hospital, 900 Commonwealth Avenue, Boston, MA 02215, USA; ^2^School of Engineering and Applied Sciences and East Asian Programs, Harvard University, Harvard Yard, Cambridge, MA 02138, USA; ^3^KP Technology Ltd., Wick KW1 5LE, UK; ^4^Department of Physics, University of Oslo, 0316 Oslo, Norway; ^5^Department of Biomedical and Clinical Engineering, Rikshospitalet University Hospital, Oslo University Hospital, 0027 Oslo, Norway; ^6^Departments of Dermatology & Ophthalmology, Research Institute for Regenerative Cures, UC Davis School of Medicine, 2921 Stockton Boulevard, Sacramento, CA 95817, USA; ^7^School of Medical Sciences, University of Aberdeen, Aberdeen AB25 2ZD, UK; ^8^Martinos Center for Biomedical Imaging, Department of Radiology, Massachusetts General Hospital, 149 Thirteenth Street, Charlestown, MA 02129, USA; ^9^Division of General Medicine & Primary Care, Department of Medicine, Beth Israel Deaconess Medical Center, 330 Brookline Avenue, Boston, MA 02215, USA

## Abstract

*Objective*. Acupuncture points are reportedly distinguishable by their electrical properties. However, confounders arising from skin-to-electrode contact used in traditional electrodermal methods have contributed to controversies over this claim. The Scanning Kelvin Probe is a state-of-the-art device that measures electrical potential without actually touching the skin and is thus capable of overcoming these confounding effects. In this study, we evaluated the electrical potential profiles of acupoints LI-4 and PC-6 and their adjacent controls. We hypothesize that acupuncture point sites are associated with increased variability in potential compared to adjacent control sites. *Methods*. Twelve healthy individuals were recruited for this study. Acupuncture points LI-4 and PC-6 and their adjacent controls were assessed. A 2 mm probe tip was placed over the predetermined skin site and adjusted to a tip-to-sample distance of 1.0 mm under tip oscillation settings of 62.4 Hz frequency. A 6 × 6 surface potential scan spanning a 1.0 cm × 1.0 cm area was obtained. *Results*. At both the PC-6 and LI-4 sites, no significant differences in mean potential were observed compared to their respective controls (Wilcoxon rank-sum test, *P* = 0.73 and 0.79, resp.). However, the LI-4 site was associated with significant increase in variability compared to its control as denoted by standard deviation and range (*P* = 0.002 and 0.0005, resp.). At the PC-6 site, no statistical differences in variability were observed. *Conclusion*. Acupuncture points may be associated with increased variability in electrical potential.

## 1. Introduction

One fundamental question remains largely unaddressed in acupuncture research: what is an acupuncture point? The answer to this question carries substantial implications for research and determines the appropriateness of a sham control, the rationale for employing various techniques (e.g., electrical stimulation and magnets), and the optimal point localization techniques for animal models. The proper characterization of acupuncture points is arguably as critical to acupuncture research as quality assurances are to botanical research, yet neither researchers nor clinicians have fully arrived at a consensus on how acupuncture points should be defined or localized. 

In the acupuncture community, acupuncture points have traditionally been viewed as points of distinct electrical characteristics [1]. This view dates to the 1950s, when Voll (Germany) in 1953 [2], Nakatani (Japan) in 1956 [3, 4], and Niboyet (France) in 1957 [5] independently concluded that skin points with unique electrical characteristics were identifiable and spatially correlated with traditional acupuncture points. Since then, a number of studies have elaborated the electrical properties attached to these “bioactive” points and ascribed these points with increased conductance [6–8], reduced impedance and resistance, increased capacitance [9–14], and elevated electrical potential compared to nonacupuncture points [8, 15–17].

For sixty years, these claims have remained unsettled due, in large part, to confounders inherent to electrodermal devices relying on electrodes contacted with skin. Confounding factors—namely, electrode pressure, choice of contact medium, electrode polarization, and skin moisture—collectively contribute to measurement variability and susceptibilities to bias [18]. To overcome these issues, we have employed a novel Scanning Kelvin Probe to measure surface electrical potential without actually touching the skin, and this study represents the first time, to our knowledge, where this technology has been applied to the study of acupuncture points in an *in vivo* human setting. The Scanning Kelvin Probe relies on capacitive coupling between the probe and the sample and has been used in metal work function determination [19–21], dopant profile characterization in semiconductor devices [22–26], metal corrosion analysis [19, 27], and liquid-air interface characterization [28, 29] with micrometer scale and millivolt resolution. The theoretical basis for applying this technology to biological tissue has been published elsewhere [30].

 In this study of 12 healthy subjects, we obtained 1.0 cm × 1.0 cm scans of surface potential over acupuncture points LI-4 and PC-6 and their respective, adjacent controls. We hypothesized that scans of and around the acupuncture point are associated with increased topographic variability in electrical potential compared to the scans of adjacent controls. This hypothesis was derived from the theoretical idea that acupuncture points are electrophysiologically distinct from their adjoining skin and thus engender greater spatial variability in electrical potential for a region encompassing both acupoint and its vicinity. 

## 2. Materials and Methods

### 2.1. Scanning Kelvin Probe: Setup

**Figure 1:**
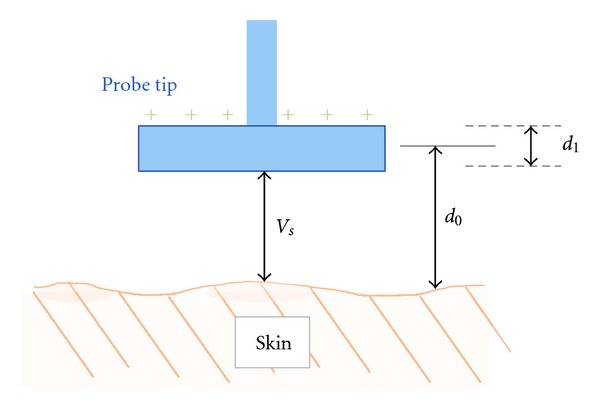
Illustration of the Scanning Kelvin Probe arrangement. The Kelvin Probe tip is maintained over the skin at a distance of *d*_0_ to create a capacitor arrangement. Due to the intrinsic potential differences between the tip and the skin (*V*_*S*_), charges accumulate at the tip once a closed circuit is established. The tip oscillates at an amplitude of *d*_1_ which generates a current through the Scanning Kelvin Probe circuit.

The Scanning Kelvin Probe (SKP5050, Kelvin Probe Technology, Ltd., Wick, UK) is a state-of-the-art device that measures surface electrical potential without actually contacting the sample [31]. Its operation can be grossly summarized as follows (Figure 1): a probe tip is positioned close to the skin, creating a capacitor; the probe tip acts as a plate while the skin acts as the contralateral plate and the potential difference between the two (*V*_*S*_) generates a charge on the probe tip; the probe tip oscillates to vary the distance from the skin (*d*_0_: tip-to-sample distance, 2*d*_1_: probe oscillation amplitude); since capacitance is inversely related to distance, the oscillation changes the capacitance and alters the charge on the probe tip; this generates a measurable current (approximately around 10^−9^ amperes) which is used to calculate the potential difference between the tip and sample; with a constant work function seen with the metallic tip, the skin surface potential can be determined. Our Scanning Kelvin Probe has the added capabilities of (1) calculating the tip-sample distance within an accuracy of ~1 *μ*m and (2) scanning the surface to produce a two-dimensional potential profile [32, 33].

The probe tip is circular, 2 mm in diameter, and composed of stainless steel. Preliminary studies revealed that biological potential measurements with the steel tip were not sensitive to modest shifts in temperature (±10°F) or humidity (±5%). The tip oscillated at a frequency of 62.4 Hz and an amplitude of 70 *μ*m. The probe tip was set at a constant “gradient” of 210 corresponding to a tip-to-sample distance of approximately 1.0 mm. The “gradient” is a Kelvin Probe measurement that is inversely proportional to the distance squared and is derived from applying a variable backing potential to the tip. A detailed description of this parameter and its derivation is described elsewhere [30, 31]. Data was acquired at a rate of 13,500 Hz, gain of 5, and averaging of 10 to extract the surface electrical potential as previously described [31]. 

Because the Kelvin Probe is very sensitive to ambient electric fields, a Faraday cage composed of fine copper mesh (16-mesh, TWP Inc., Berkeley, CA) was fabricated and used to enclose the Kelvin Probe head unit and automatic motor scanner unit, along with the subject's hands and wrists. All conductive materials within the Faraday cage were grounded to an isopotential level using conducting wires connected to a central grounding unit. Insulating materials were either removed or, if required, sprayed with antistatic spray. Furthermore, testing was completed in an electrically shielded room located within the CRC Biomedical Imaging Core at the MGH Charlestown campus. The complete Kelvin Probe unit was rested on a large 30′′ × 36′′ Vibration Isolation Workstation (KSI Model number 910R-01-45, Kinetic Systems Inc., Boston, MA) to minimize noise arising from mechanical disturbances. 

### 2.2. Recruitment

 Twelve healthy subjects (5 females, 7 males) were recruited to participate in the study. Participants were recruited via postings in Craigslist (http://www.craigslist.org). “Healthy” was defined as absence of a chronic medical condition requiring daily medications (e.g., hypertension, diabetes, hypothyroidism, etc.). Individuals with autonomic disorders (sweating irregularities), skin disorders, extensive burns/scars on the hand, tremors, neuromuscular conditions, restless leg syndrome, movement disorders, and implanted cardiac defibrillator/pacemaker were excluded. The subjects' mean age was 33.7 ± 9.8 (±SD) years. Demographic representation was 7 non-Hispanic White, and 5 Asian.

This study was reviewed and approved by the Institutional Review Board at Partners Healthcare. Each study participant read and signed an informed consent form.

### 2.3. Scanning Measurements

Study volunteers were asked to sit motionless while their wrist and hand were secured with grounding straps to the optical breadboard that served as the base for the Kelvin Probe unit. Because hair may interfere with voltage measurements, each tested site was previously naired to remove all hair within the region. A silver/silver chloride strip electrode (EL-506, Biopac Inc., Goleta, CA) with conductive electrode gel was placed on the ulnar aspect of the forearm approximately 5 cm proximal to the wrist joint. This electrode served as both ground and reference electrode and was intentionally placed close to the test sites to minimize incorporation of physiological electrical activity (e.g., muscle or electrocardiographic) arising from the intervening spaces. 

The arm was placed either in a supinated or pronated position depending on the site being evaluated. The hand or wrist was positioned in a way that would keep the surface as flat as possible with respect to the Kelvin Probe tip. In each of the 12 subjects, two acupoints, LI-4 and PC-6, and their corresponding control points were tested. LI-4 was located on the dorsum of the hand, between the first and second metacarpal bones, at the midpoint of the second metacarpal bone and close to its radial border [34]. Its control was exactly 1 cm ulnar to LI-4. PC-6 was located on the flexor aspect of the forearm, 2 cun (a unit of proportional measurements used in acupuncture practice) proximal to the wrist crease and between the tendons of palmaris longus and flexor carpi radialis [34]. Its control was located at either 1 cm radial (7 subjects) or 1 cm ulnar to the point (5 subjects). The radial control was employed for the first seven subjects but switched subsequently to the ulnar control after realizing that the radial control coincided with Japanese-style localization of PC-6. The order of testing by laterality (left versus right), test region (dorsum of the hand versus volar aspect of forearm), and point classification (acupuncture point versus control) was randomized.

Once an acupuncturist identified the points, the corner edges of a 1.0 cm × 1.0 cm square region were marked, centered over the point. The tip was placed over one of the corners and scanning was performed sequentially by rows. The probe was moved with 2 mm intervals to create a 6 × 6 topographic matrix of the surface electrical potential. At each point, a total of 50 electrical potential measurements were acquired continuously to optimize the signal-to-noise ratio, corresponding to a standard error of 6–8 mV per point. After obtaining 50 measurements at a point, the tip was subsequently moved over an adjacent scan point to acquire another set of measurements. These potential measurements were acquired under a “Tracking” algorithm where data were only recorded within a specified range of “gradient,” a marker for probe-to-sample distance. The SKP5050 was equipped with a vertical motor that automatically corrected for any deviations from the desired gradient. Each scan of a test site took approximately 20–25 minutes to perform, corresponding to approximately 35 seconds over each point. 

### 2.4. Calculations and Statistical Analyses

Topographic maps of electrical potentials were obtained by averaging the 50 electrical potential measurements associated with each matrix point. The maps were displayed as a 3-D surface map using Matlab (version 2011b, Mathworks, Natick, MA) to identify any overall electrical potential patterns. In some instances, a consistent elevation or decrease (greater than 50–100 mV) in electrical potential was identified at a matrix point and correlated with subjective sensations of light touch and with the existence of small hairs incompletely removed by Nair. These data points were removed from analyses. 

The mean, standard deviation, and range (highest minus smallest potential value) of electrical potential measurements associated with each square scan were calculated, and the Wilcoxon rank sum-tests (Matlab 2011b) were performed to evaluate differences in these variables between acupuncture points and their respective controls. 

## 3. Results

**Figure 2:**
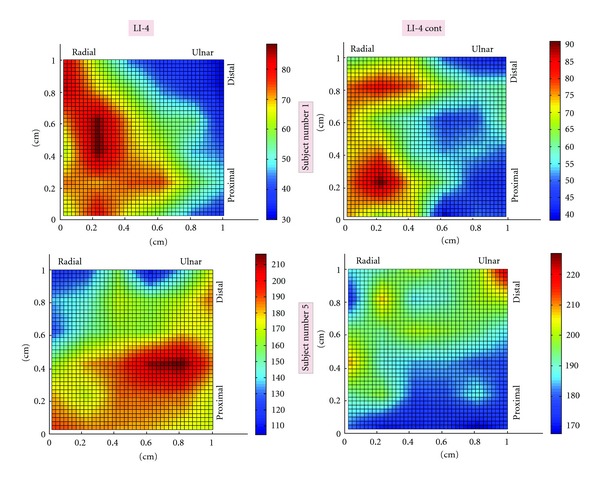
Topographic maps of electrical potential at LI-4 and control sites. Representative topographic maps from two subjects are shown here. Images on the left correspond to LI-4 while the images on the right correspond to LI-4 Control. The top images are derived from Subject number 1 and the bottom images are from Subject number 5. For each scan, a color bar is included to display electrical potential magnitudes.

**Figure 3:**
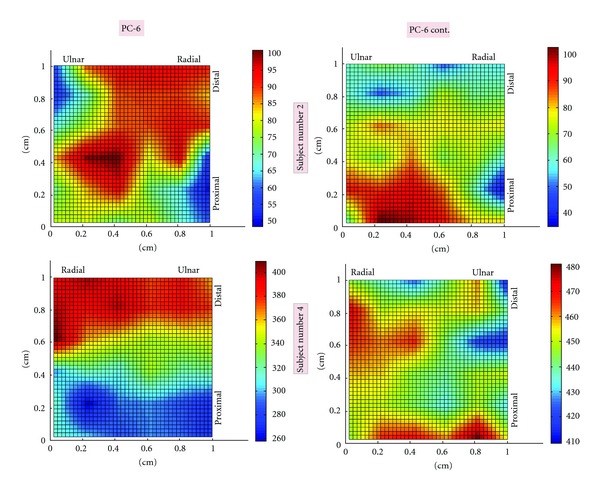
Topographic maps of electrical potential at PC-6 and control sites. Representative topographic maps from two subjects are shown here. Images on the left correspond to PC-6 while the images on the right correspond to PC-6 control. The top images are derived from Subject number 2 and the bottom images are from Subject number 4. For each scan, a color bar is included to display electrical potential magnitudes.

Representative topographic scans of electrical potentials at LI-4 and their corresponding adjacent controls are displayed in Figure 2.  Figure 3 shows representative scans of PC-6 and their respective controls. Although a single, coherent peak in potential is seen in several LI-4 topographic scans, no such clear-cut patterns were seen at other sites—including PC-6. 

As seen in Table 1, the *mean* potentials at LI4 and PC6 sites were not statistically different from their respective controls (Wilcoxon rank-sum, *P* = 0.73 and 0.79, resp.). The variability in electrical potential—as evident by both the *standard deviation *and the *range*—was significantly increased at LI-4 site compared to its control (*P* = .002 and 0.0005, resp.). Except for one subject, every tested individual had greater *standard deviation* in potential at LI-4 site compared to its control, whereas all individuals were found to have greater *range* in electrical potential at LI-4 sites. At PC-6 and PC-6 control sites, on the other hand, no statistical differences in variability were observed (*P* = 0.27 for standard deviation and *P* = 0.20 for range). The location of PC-6 controls (radial versus ulnar) had no effect on the study results as no differences in potential variability were seen in either comparisons.

In general, the Scanning Kelvin Probe revealed a not insignificant amount of spatial variability in electrical potential within each 1 cm^2^ area. The average difference between the highest and lowest potential within each site was 50 to 80 mV and was found to be as large as 150 mV at some sites. 

## 4. Discussion

This is the first study, to our knowledge, where the electrical properties of acupuncture points were evaluated using a noncontact method. Our approach differs from previous studies in two fundamental ways: first, electrical measurements were obtained without the requirement of an active skin electrode, and second, the Kelvin Probe measures electrical *potential* in contrast to the more common electrical *impedance* acquired in other electrodermal studies. These distinctions are associated with several notable advantages and disadvantages. 

By obtaining electrical potential without contacting the sample, the Scanning Kelvin Probe bypasses the electrode-skin confounders that plague most, if not all, existing electrodermal devices. The Kelvin Probe is not limited by variable ion accumulation at the electrode, microscopic irregularities of the electrode surface, the effects of contact medium, the variability in mechanical pressure, or the influence of stratum corneum moisture on electrical measures. Moreover, by hovering over the skin surface, the probe tip is capable of scanning the area using a motorized raster unit while maintaining a steady tip-to-sample distance with *μ*m resolution based on a validated Baikie method [31, 33].

However, by virtue of its noncontact approach, the Kelvin Probe is also susceptible to ambient field effects and movement artifacts. Our apparatus involved an electrically shielded room, a local Faraday cage, electrical grounding of all proximate conductive material, strapping of the hand and wrist to the base board, and a large vibration isolation workstation to attenuate any mechanical perturbations. Even under such controlled conditions, the signal-to-noise ratio was such that numerous potential measurements at each matrix point were required to obtain a sufficiently precise measurement for the purpose of this study. As a consequence, each topographic scan required at least 20 minutes of recording to be completed. In that interim, the wrist and hand could have unwittingly moved and, in few subjects, displaced as much as 6 mm in either longitudinal or lateral directions (although most individuals were able to maintain the position within a 2 mm range). 

The volar aspect of the wrist—PC-6 site and its control— in particular, was prone to these movement artifacts since the fully supinated position was more difficult to maintain than the pronated position and the region was frequently traversed by superficial veins that led to slower acquisition of data (interestingly, the respiratory and cardiac mechanical pulsations in the veins could be observed with the Kelvin Probe). These factors may account for why the PC-6 site did not demonstrate a statistical difference compared to its neighboring control. Temporal changes in skin potential over the 20 minute interval may also account for our study results, although ongoing studies with a larger 5 mm tip have demonstrated no substantial change in surface potential over a 40-minute period. 

Acquisition of surface electrical potential with the Kelvin Probe has a number of advantages. Without relying on intercalating dyes, strong electrical fields, ionizing beams, or penetrating needles, the Kelvin Probe is well-suited for *in vivo* use. Rather than perturbing the system with substantial electrical currents, as is done in most electrical impedance approaches, surface potential techniques, such as the Scanning Kelvin Probe, introduce little-to-no current and therefore have the theoretical capacity to capture the native and uninhibited endogenous functions of the body. For these reasons, it is not surprising that prior attempts have been made to measure electrical potentials on and around acupuncture points. A total of four studies within the English literature reported that the electrical potential at acupuncture points were, on average, 5 to 100 mV more positive than adjacent skin areas [8, 16, 17, 35]. The non-English literature also agreed with this relative direction in potential [17, 36]. Our study, in contrast, identified no such consistent relationship and found no statistical differences between mean potentials at acupuncture point and adjacent control sites. Importantly, these prior studies were largely anecdotal in nature and did not have control sites, did not perform statistical analyses, and did not account for skin-electrode factors—such as ionization and redox potentials—that can still confound potential measurements. 

The functional significance of the increased variability in electrical potential at LI-4 sites is unclear. Unlike electrical impedance, the physiological factors underlying skin potential measurements have not been fully elaborated and present a significant limitation in our ability to interpret the data. Recent advances in wound healing research, however, have provided some important insights by revealing that a transepithelial potential gradient exists in both amphibian and mammalian skin [37]. Sodium and potassium ions are selectively transported by ion pumps to the inner extracellular layer (i.e., dermal side of the epidermis), while chloride ions are passively transported to the external surface of the skin [38, 39]. This charge separation generates a transepithelial electrical potential that is maintained by apical tight junctions between the outer epidermal cells. In mammalian skin, this transepithelial potential is approximately 70 mV in magnitude—the outer epidermis being more electronegative compared to inner epidermis [40]. Interestingly, this is within the same magnitude of potential changes seen across a 1 cm^2^ span of skin, and it is conceivable that variations in ion pump activities within the epidermis can account for the increased spatial variability in potential seen at LI-4 sites. Certainly, topical applications of pump and channel inhibitors (e.g., amiloride and tetrodotoxin) can be used in future studies to test this hypothesis [41]. 

This study has a number of limitations. First, as previously stated, the Kelvin Probe is sensitive to ambient field and physical movement artifacts, and potential measurements were affected by superficial structures such as hairs and subcutaneous veins. Second, the prolonged scan time for each site may predispose the topographic map to a number of unintended effects, including lateral displacement of the hand/wrist, changes in local circulation, and subject fatigue. Third, our decision to utilize 2 mm tips and to scan 1.0 cm × 1.0 cm area may be either too small or too large for the purposes of evaluating an acupuncture point. Future studies should consider evaluating larger scan areas with our present tip or smaller scan areas with smaller tips. Lastly, despite using well-described anatomic landmarks for identifying acupuncture points, we may have incorrectly identified the location of the acupuncture points. Although the scan area provides some level of flexibility, it is worth noting that the exact locations of PC-6 and LI-4 acupuncture points, themselves, are still to some degree disputed. Some of our PC-6 control sites, for instance, can be arguably located on the Japanese-acupuncture-defined PC-6. 

 Despite these limitations, this study identified a nearly universal increase in variability of potential at LI-4 site compared to its control and provided, for the first time, data on the spatial distribution of *in vivo* electrical potential on intact human skin using a noncontact approach. Future Kelvin Probe studies may consider evaluating the temporal variability of the electrical potential and the electrical field strength over acupuncture points and corresponding controls. 

## 5. Conclusion

The Scanning Kelvin Probe revealed no differences in average electrical potential between acupuncture point and adjacent control sites, but showed a significant increase in variability at the LI-4 area compared to its adjacent control. No such differences were seen at PC-6. The Scanning Kelvin Probe is a promising, novel technology for evaluating *in vivo* skin potentials. Although this application of the Scanning Kelvin Probe is in its early stages, future advances may help yield important insights about the nature of acupuncture points. 

## Figures and Tables

**Table 1:** Topographic characteristics of electrical potential scans.

Location	Scan parameters		
	Mean (mean ± SE, mV)	Standard deviation	Range (mean ± SE, mV)
Dorsal hand			
LI-4	135.1 ± 24.2	18.7 ± 1.8	80.8 ± 9.2
LI-4 control	139.0 ± 24.8	12.5 ± 0.9	52.7 ± 4.8
*P* value	0.73	**0.002**	**0.0005**
Volar wrist			
PC-6	138.1 ± 29.3	16.2 ± 2.8	66.0 ± 9.2
PC-6 control	138.4 ± 34.8	17.4 ± 1.7	76.1 ± 7.9
*P* value	0.79	0.27	0.20
